# Fulminant Pneumococcal Meningitis With Neurovascular Complications in an Adult With Sickle Cell Anemia: A Case Report

**DOI:** 10.7759/cureus.100042

**Published:** 2025-12-24

**Authors:** Saed Omer, Ibrahim Alnashri, Hassan Alzahrani, Malek Almalki, Ahmed Alsum, Abdulrahman Mohanna, Khairallah Belkhouja

**Affiliations:** 1 Intensive Care Unit, King Fahad Armed Forces Hospital, Jeddah, SAU; 2 Radiology, King Fahad Armed Forces Hospital, Jeddah, SAU

**Keywords:** cerebrospinal fluid, cerebrovascular complications, frank pus, pneumococcal meningitis, saudi arabia, sickle cell anemia, vaccination

## Abstract

We report a 45-year-old woman with sickle cell anemia (SCA) who presented with fever, severe headache, and decreased level of consciousness. Initial brain computed tomography (CT) was unremarkable. Diagnostic lumbar puncture (LP) revealed the uncommon, frankly purulent cerebrospinal fluid (CSF) with profound neutrophilic pleocytosis, markedly elevated protein, undetectable glucose, and polymerase chain reaction (PCR)/culture confirmation of Streptococcus pneumoniae. Empiric therapy with ceftriaxone, vancomycin, dexamethasone, and acyclovir was initiated. Within 72 hours, brain magnetic resonance imaging (MRI) and CT demonstrated multifocal infarcts, basal ganglia hemorrhage, and malignant cerebral edema. Despite intensive neurocritical care, she progressed to brain death. This case highlights the rare, grossly purulent CSF phenotype in adult pneumococcal meningitis and the rapid development of cerebrovascular complications in patients with SCA. Clinicians should maintain a low threshold for meningitis, initiate prompt empiric therapy, escalate neurocritical monitoring for early complications, and ensure up-to-date pneumococcal vaccination in high-prevalence regions and among high-risk patients.

## Introduction

Sickle cell anemia (SCA) is the most common hemoglobinopathy worldwide and predisposes patients to lifelong susceptibility to invasive infections caused by encapsulated bacteria. Functional asplenia or hyposplenism, impaired opsonization, and altered complement activity weaken IgM-memory B-cell responses and reduce clearance of polysaccharide-encapsulated organisms such as Streptococcus pneumoniae [1,2]. In Saudi Arabia, SCA and sickle cell trait (SCT) exhibit marked regional variation. Premarital and community-based screening programs report an adult prevalence of approximately 0.26% for SCA and about 4% for SCT, with the highest burdens in the Eastern Province and the southwestern regions, including Jazan and Al-Ahsa/Hofuf - a pattern also reflected in newborn screening results [3-5].

Bacterial meningitis remains a critical medical emergency. Although fever, neck stiffness, and altered mental status are classic features, presentations vary, and the full triad is often absent. Globally, more than one million cases occur annually, with S. pneumoniae being the leading pathogen in adults. Individuals with sickle cell disease (SCD) face a more than 300-fold increased risk of bacterial meningitis, and mortality can approach 20% even in advanced care settings, with many survivors experiencing neurological sequelae [1,2,6,7]. In Saudi Arabia, meningitis is now infrequently detected among febrile SCD admissions, reflecting the nation’s low annual meningococcal incidence - approximately seven cases per year - and the impact of widespread vaccinations [8-10]. Nevertheless, cerebrovascular complications affect 25-31% of adults, ranging from ischemic stroke and intracerebral hemorrhage to venous sinus thrombosis and subarachnoid hemorrhage.

Timely management is essential, as delays in antimicrobial therapy significantly worsen outcomes. This illustrates the importance of immediate empiric antibiotics with adjunctive dexamethasone when bacterial meningitis is suspected. Clinicians in regions with a high prevalence of SCA should maintain a low threshold for suspecting pneumococcal meningitis in febrile or encephalopathic patients and ensure adherence to adult pneumococcal vaccination schedules [11-13]. In bacterial meningitis, cerebrospinal fluid (CSF) is often cloudy or turbid, but frankly purulent, pus-like CSF is uncommon. Diagnostic accuracy improves with rapid multiplex CSF polymerase chain reaction (PCR) platforms, such as FilmArray/BioFire, which remain reliable even after prior antibiotic exposure [14-20]. Neuroimaging further supports diagnosis: early CT may appear normal, whereas contrast-enhanced MRI-with post-contrast T1, FLuid Attenuated Inversion Recovery (FLAIR), Diffusion-weighted imaging/Apparent Diffusion Coefficient (DWI/ADC), and Susceptibility-weighted imaging (SWI) sequences-offers greater sensitivity for detecting leptomeningeal enhancement, ventriculitis, and ischemic or hemorrhagic complications [21]. Current national surveillance indicates low bacterial meningitis rates due to successful vaccination programs; however, key pathogens persist, and vigilance remains essential during mass gatherings such as Hajj and Umrah [8-10].

## Case presentation

A 45-year-old woman with SCA and hypothyroidism, whose vaccination history was unknown, presented with a two-day history of sore throat, headache, fever, and altered mental status. Two weeks earlier, she had undergone an abortion followed by uterine dilation and curettage. She was initially evaluated at another hospital, diagnosed with an upper respiratory tract infection and a vaso-occlusive pain crisis, and discharged on oral antibiotics. A worsening headache, persistent fever, and progressive confusion then prompted her presentation to our emergency department.

On examination, she appeared ill and tachycardic, with a blood pressure of 140/70 mmHg, a heart rate of 115 beats per minute, a respiratory rate of 28 breaths per minute, a temperature of 38.7°C, and an oxygen saturation of 95% on room air. Her Glasgow Coma Scale (GCS) was 9/15: she opened her eyes to pain, produced incomprehensible sounds, and localized to a sternal rub. Pupils were 4 mm and reactive. Corneal, vestibulo-ocular, and gag reflexes were intact. She exhibited nuchal rigidity with positive Brudzinski and Kernig signs.

Laboratory tests showed leukocytosis, anemia, and markedly elevated CRP and procalcitonin levels. Electrolytes and renal function were unremarkable (Table 1).

**Table 1:** Laboratory investigations BNP: B-type Natriuretic Peptide; ALT: Alanine transaminase; AST: Aspartate transaminase; GGT: Gamma-glutamyltransferase; INR: International Normalized Ratio.

Test	Result	Ref (typical adult)	Interpretation
White blood cell count	12,700 /µL	4,000–11,000 /µL	High (leucocytosis)
Haemoglobin	90.8 g/L	120–155 g/L (female)	Low (anaemia)
CRP	269 mg/L	<10 mg/L	Very high (inflammation/infection)
Procalcitonin	33.6 ng/mL	<0.5 ng/mL	Markedly elevated (bacterial range)
Sodium	147 mmol/L	135–145 mmol/L	Slightly high
Potassium	3.0 mmol/L	3.5–5.0 mmol/L	Low
Lactate	1.7 mmol/L	0.5–2.0 mmol/L	Normal
Troponin (assay not specified)	103 ng/L	Assay‑specific; typical 99th centile ~14 ng/L	Elevated
BNP	570 pg/mL	<100 pg/mL (assay‑dependent)	Elevated
Total bilirubin	85 µmol/L	5–21 µmol/L	High
ALT	86 U/L	<35 U/L	High
AST	61 U/L	<35 U/L	High
GGT	100 IU/L	<60 IU/L	High
INR	1.35	0.9–1.1	Slightly high
Platelets	Within normal range (value not recorded)	150–400 ×10³/µL	Normal (reported)
Renal function	Within normal range (values not provided)	—	Normal (reported)

**Figure 1:**
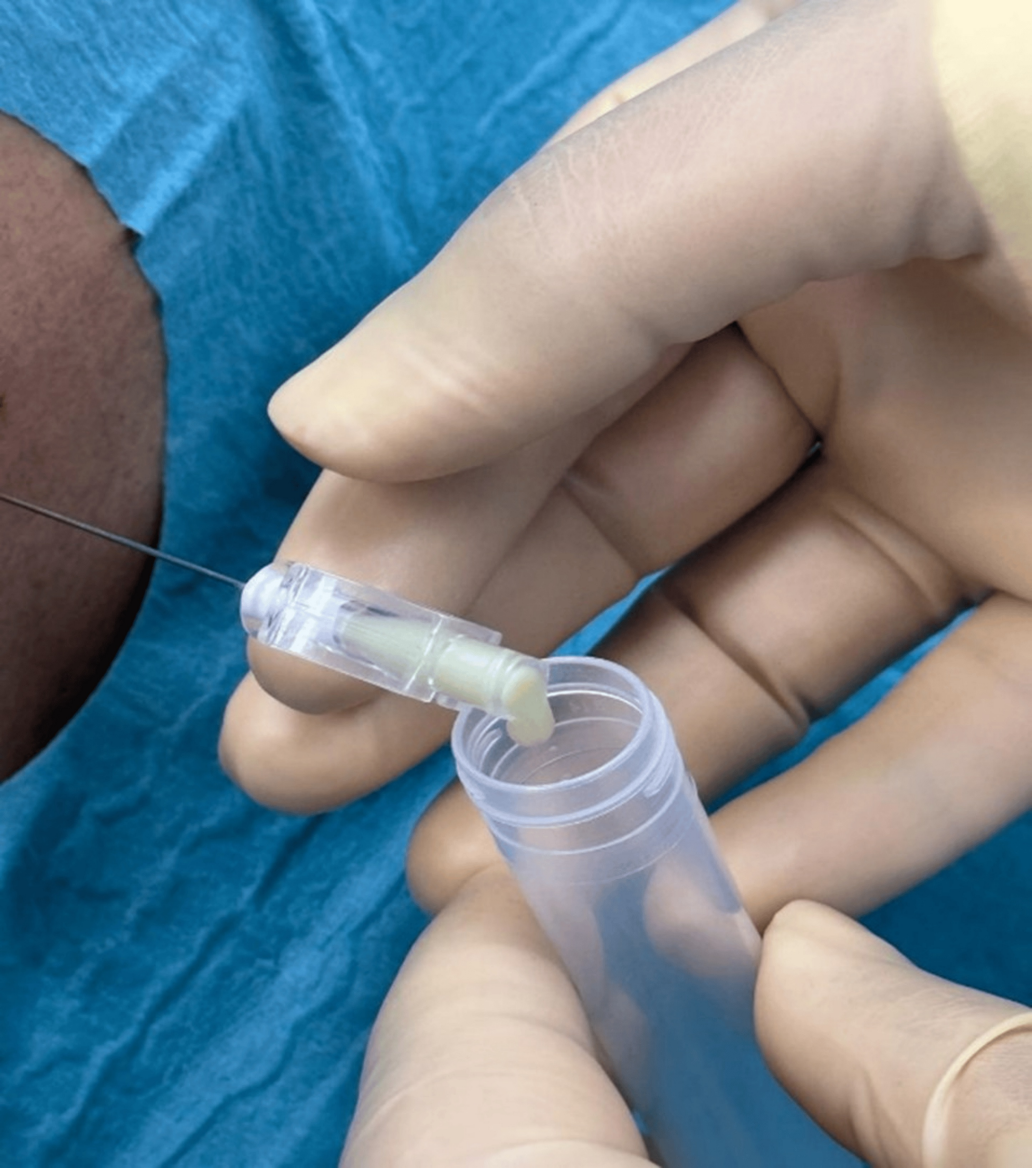
Lumbar puncture (LP) Cerebrospinal fluid (CSF) appears thick and whitish.

Non-contrast head CT was normal. Lumbar puncture revealed frankly purulent, whitish-yellow CSF resembling pus (Figure 1).

CSF analysis showed a markedly elevated white blood cell count, very high protein, severely low glucose, and negligible red blood cells (Table 2).

**Table 2:** CSF indices at lumbar puncture CSF: Cerebrospinal fluid; WBC: White Blood Cell; RBC: Red Blood Cell; PCR: Polymerase chain reaction.

CSF parameter	Result	Typical reference	Interpretation
Appearance	Thick, whitish‑yellow; frankly purulent (“pus-like”)	Clear/colourless	Purulent
Opening pressure	Not recorded	6–25 cm H₂O (adult)	—
WBC	48,600 /µL	<5 /µL	Marked neutrophilic pleocytosis
RBC	<1 /mm³ (≈ <1 /µL)	<5 /µL	Minimal RBCs (non-traumatic tap)
Protein	12.66 mg/mL (≈ 1,266 mg/dL)	15–45 mg/dL (0.15–0.45 g/L)	Extremely high
Glucose	<0.06 mmol/L (≈ <1.1 mg/dL)	2.5–4.4 mmol/L (~45–80 mg/dL)	Very low
Microbiology	Streptococcus pneumoniae detected (multiplex PCR & culture)	Negative	Pathogen identified

Her venous blood gas revealed alkalosis with mildly decreased pCO₂, increased HCO₃⁻, and low pO₂ (Table 3).

**Table 3:** Venous blood gas parameters

Parameter	Result	Ref (venous)	Interpretation
pH	7.5	7.31–7.41	Alkalemia
pCO₂	35 mmHg	40–50 mmHg	Low
pO₂	54 mmHg	~30–40 mmHg^†^	Higher than usual venous; ^†^limited interpretative value for oxygenation assessment
HCO₃	27 (unit as recorded)	22–28 mmol/L	Normal

Multiplex PCR and CSF culture identified Streptococcus pneumoniae. Empiric intravenous ceftriaxone, vancomycin, dexamethasone, and acyclovir were initiated, and the patient was transferred to the ICU.

**Figure 2:**
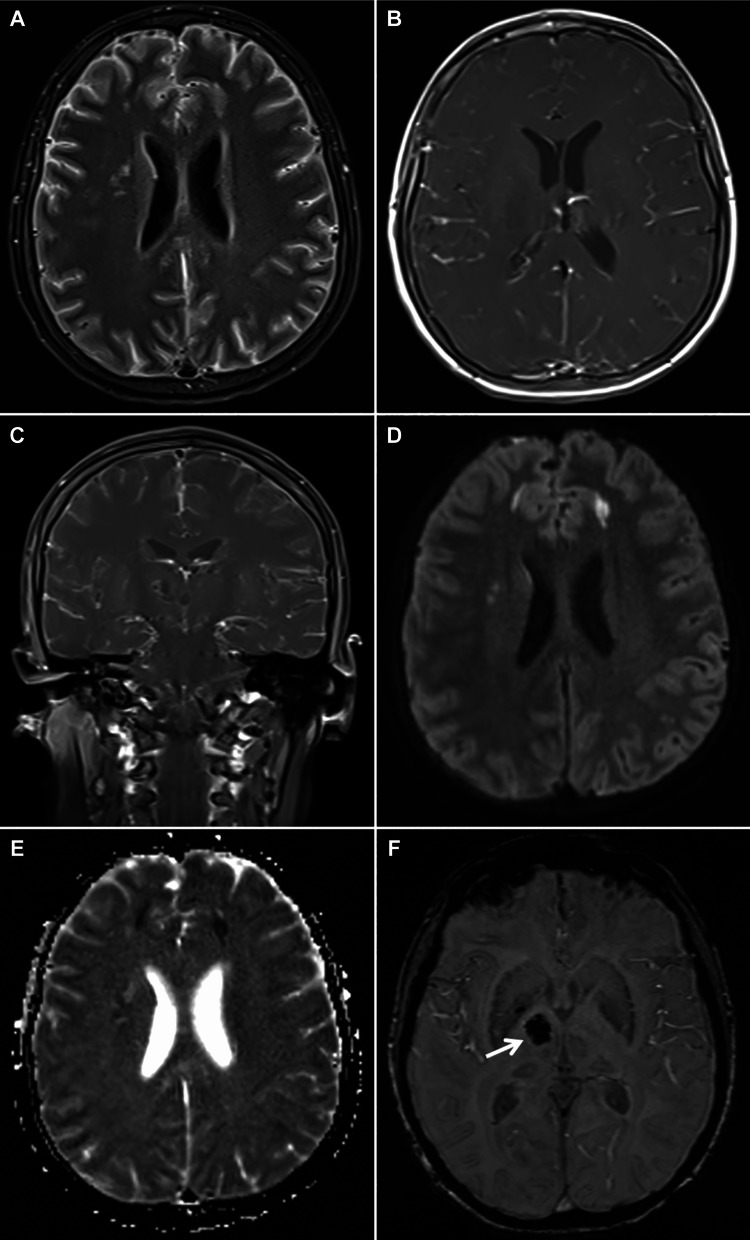
Brain MRI with gadolinium (A) Axial Fluid attenuated inversion recovery (FLAIR) image showing leptomeningeal enhancement. (B) Axial T1-weighted image showing leptomeningeal enhancement. (C) Coronal T1-weighted image revealing leptomeningeal enhancement. (D) Axial diffusion-weighted imaging (DWI) displaying bilateral frontal ischemic insults. (E) Axial apparent diffusion coefficient (ADC) map indicating bilateral frontal ischemic insults, confirmed by correlation with the DWI. (F) Axial susceptibility-weighted imaging (SWI) showing right basal ganglia hemorrhage. MRI, magnetic resonance imaging.

On day two, due to a persistently low GCS, she underwent elective endotracheal intubation and contrast-enhanced MRI, which demonstrated diffuse leptomeningeal enhancement and multiple bilateral cortical and subcortical diffusion-restricted lesions consistent with acute infarctions. SWI revealed a right basal ganglia hemorrhage (Figure 2).

On day three, repeat CT showed disease progression, with cortical and deep white-matter hypodensities in both frontal and parietal lobes, as well as in the left occipital and temporal cortex, indicating acute ischemic insults. A right basal ganglia/thalamic intraparenchymal hemorrhage with surrounding edema exerted mass effect on the third ventricle and right ambient cistern, raising concern for impending uncal herniation. Mannitol was initiated for intracranial hypertension.

**Figure 3:**
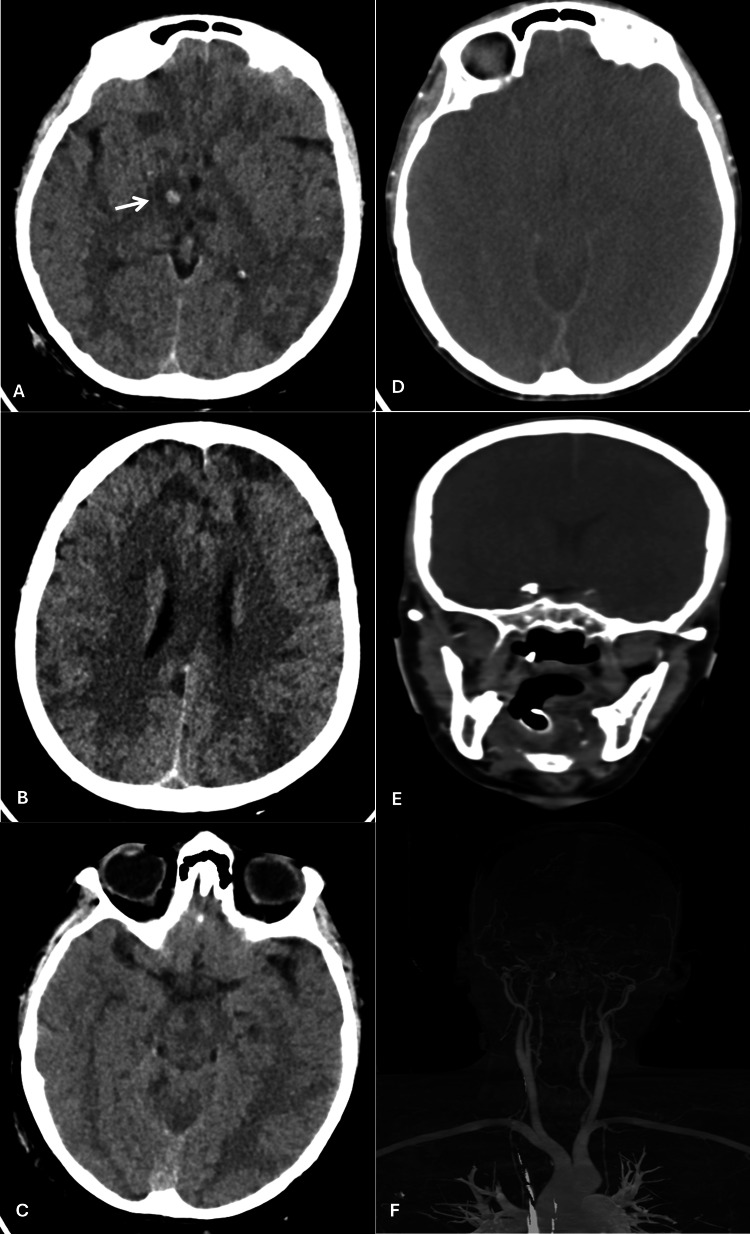
Computed tomography (CT) of the brain (A) Axial non-contrast CT showing a right basal ganglia hemorrhage with surrounding edema, causing effacement of the third ventricle. (B) Axial non-contrast CT revealing bilateral frontal hypodensities consistent with ischemic insults. (C) Axial non-contrast CT demonstrating impending uncal herniation. (D) Axial CT perfusion scan showing diffuse cerebral edema. (E) Coronal CT perfusion scan revealing non-opacification of intracranial vessels. (F) Coronal CT perfusion scan with subtraction imaging demonstrating non-opacification of intracranial vessels.

By day five, her GCS had deteriorated to 3/15, with absent brainstem reflexes and a positive apnea test. Cerebral perfusion imaging demonstrated widespread cerebral edema and non-opacification of intracranial vessels, confirming brain death (Figure 3).

Despite supportive care, she died in the ICU.

## Discussion

This case demonstrates fulminant pneumococcal meningitis in an adult with SCA, highlighting the rare macroscopic finding of visible pus in the CSF and the rapid progression to multifocal infarction, intracerebral hemorrhage, malignant cerebral edema, and brain death. The predisposition of patients with SCA to severe infections results from functional asplenia or hyposplenism, impaired opsonization, and complement dysregulation. Despite the availability of conjugate vaccines, adults with SCD remain at substantially increased risk for invasive pneumococcal disease and tend to experience more severe illness than the general population [1,2].

Bacterial meningitis still carries significant mortality and disability even with prompt treatment. The timing of antimicrobial administration is critical; delays are associated with stepwise increases in mortality and neurological complications. Empiric antibiotics and dexamethasone should be administered immediately when meningitis is suspected. Neuroimaging prior to lumbar puncture is reserved for patients with focal neurological deficits, papilledema, new-onset seizures, or depressed consciousness, as in our patient, to avoid complications related to diagnostic lumbar puncture. Her rapid clinical deterioration highlights the extremely narrow therapeutic window in fulminant pneumococcal disease [18,19,22].

In Saudi Arabia, the Eastern and Southwestern regions (e.g., Al-Ahsa/Hofuf and Jazan) have the highest prevalence of SCA. When combined with the ongoing, albeit low, national rates of meningitis, these patterns justify a low threshold for meningitis evaluation and proactive preventive measures [3-5,8-13].

CSF in bacterial meningitis is usually turbid, with neutrophilic pleocytosis, elevated protein, and decreased glucose. Frankly, purulent (“pus-like”) CSF is uncommon but reported in severe pyogenic meningitis and ventriculitis, where dense intraventricular exudate and extremely high CSF leukocytosis portend poor outcomes. Rapid multiplex PCR platforms maintain diagnostic accuracy after antibiotic pretreatment and facilitate pathogen-specific therapy, especially when cultures are negative [20,23,24].

The cerebrovascular spectrum of pneumococcal meningitis includes multifocal ischemia, intracerebral hemorrhage, venous thrombosis, and subarachnoid hemorrhage [25]. Mechanisms include inflammatory vasculopathy or vasculitis, endothelial disruption from pneumolysin, coagulation activation with impaired fibrinolysis, and, rarely, infectious aneurysm formation [26]. Early hemorrhage is associated with worse outcomes [27]. Magnetic Resonance Venography (MRV) should be considered when venous thrombosis is suspected, while Computed Tomography Angiography (CTA) or Magnetic Resonance Angiography (MRA) is appropriate when aneurysm or vasculitis is a concern [21,26]. MRI with contrast provides superior sensitivity for leptomeningeal enhancement and cerebritis. DWI/ADC sequences detect early infarcts, and SWI reveals microbleeds or hemorrhage. Post-contrast FLAIR imaging may outperform post-contrast T1-weighted imaging in visualizing meningeal disease [21]. Although the initial CT scan may be normal, a normal CT should not delay the initiation of antibiotics when meningitis is suspected [19].

Empiric therapy for adults generally includes ceftriaxone (or cefotaxime) plus vancomycin and dexamethasone until susceptibilities are known, ensuring coverage for penicillin-non-susceptible pneumococci [19]. Once susceptibilities are available, therapy should be narrowed to a β-lactam alone. The usual length of treatment for pneumococcal meningitis is 10 to 14 days [19]. Dexamethasone is most effective when administered before or concurrently with the first dose of antibiotics. If pneumococcal infection is confirmed and corticosteroids were not started early, delayed initiation is not recommended [18]. Management of intracranial hypertension - including head-of-bed elevation, adequate analgesia and sedation, targeted PaCO₂ control, and hyperosmolar therapy with mannitol or hypertonic saline - is essential. Seizure control and correction of hyponatremia are also critical components of care. Although data specific to meningitis are limited, neurocritical care guidelines support the use of these interventions in cases of malignant cerebral edema [28-29]. In fulminant cases that progress to the loss of brainstem reflexes, consensus guidelines provide criteria and recommend ancillary tests, such as cerebral perfusion scintigraphy, for the determination of brain death [30].

Given the high regional burden of SCA, prevention is paramount. High-risk adults should follow vaccination schedules that include PCV20 or PCV21 once, or PCV15 followed by PPSV23 with a minimum eight-week interval. Meningococcal vaccination for Hajj and Umrah remains mandatory per national policy [8-12]. Integrating vaccine-status checks into SCD clinics in high-prevalence regions may reduce breakthrough invasive disease [3-5,11-13].

## Conclusions

Bacterial meningitis in adults with SCA can escalate rapidly, causing profound neurovascular injury over a short period. Early initiation of guideline-directed therapy - typically ceftriaxone and vancomycin with adjunctive dexamethasone, and acyclovir when encephalitis is a concern - is essential for improving outcomes. The presence of frankly purulent cerebrospinal fluid, although uncommon, signifies a severe pyogenic process that requires heightened neurocritical vigilance. Prevention remains equally essential, underscoring the need for adherence to current adult pneumococcal vaccination recommendations for sickle cell disease and full compliance with Hajj and Umrah vaccination protocols. Bacterial meningitis can lead to multifocal infarctions, basal ganglia hemorrhage, and malignant cerebral edema, ultimately progressing to brain death. This highlights the extremely narrow therapeutic window in pneumococcal meningitis associated with SCD and emphasizes the critical importance of strict adherence to established treatment guidelines.

## References

[1] Ochocinski D, Dalal M, Black LV, Carr S, Lew J, Sullivan K, Kissoon N (2020). Life‑threatening infectious complications in sickle cell disease: a concise narrative review. Front Pediatr.

[2] Halasa NB, Shankar SM, Talbot TR (2007). Incidence of invasive pneumococcal disease among individuals with sickle cell disease before and after the introduction of the pneumococcal conjugate vaccine. Clin Infect Dis.

[3] Alhamdan NA, Almazrou YY, Alswaidi FM, Choudhry AJ (2007). Premarital screening for thalassemia and sickle cell disease in Saudi Arabia. Genet Med.

[4] Memish ZA, Saeedi MY (2011). Six-year outcome of the national premarital screening and genetic counseling program for sickle cell disease and β-thalassemia in Saudi Arabia. Ann Saudi Med.

[5] Jastaniah W (2011). Epidemiology of sickle cell disease in Saudi Arabia. Ann Saudi Med.

[6] Oordt-Speets AM, Bolijn R, van Hoorn RC, Bhavsar A, Kyaw MH (2018). Global etiology of bacterial meningitis: a systematic review and meta-analysis. PLoS One.

[7] van de Beek D, de Gans J, Spanjaard L, Weisfelt M, Reitsma JB, Vermeulen M (2004). Clinical features and prognostic factors in adults with bacterial meningitis. N Engl J Med.

[8] Alhumaid NK, Alajmi AM, Alosaimi NF (2024). Epidemiology of reportable bacterial infectious diseases in Saudi Arabia. Infect Dis Ther.

[9] Badur S, Al Dabbagh MA, Shibl AM, Farahat FM, Öztürk S, Saha D, Khalaf M (2021). The epidemiology of invasive meningococcal disease in the Kingdom of Saudi Arabia: a narrative review with updated analysis. Infect Dis Ther.

[10] (2025). Invasive meningococcal disease - Kingdom of Saudi Arabia. https://www.who.int/emergencies/disease-outbreak-news/item/2025-DON563.

[11] CDC/ACIP. Pneumococcal vaccine recommendations for adults . 2024-2025 (2025). Pneumococcal Disease. https://www.cdc.gov/pneumococcal/index.html.

[12] Alharbi NS, Al-Barrak AM, Al-Moamary MS (2016). The Saudi Thoracic Society pneumococcal vaccination guidelines-2016. Ann Thorac Med.

[13] Alsaif MA, Abdulbaqi M, Al Noaim K, Aghbari M, Alabdulqader M, Robinson JL (2021). Prevalence of serious bacterial infections in children with sickle cell disease at King Abdulaziz Hospital, Al Ahsa. Mediterr J Hematol Infect Dis.

[14] Weisfelt M, van de Beek D, Spanjaard L, Reitsma JB, de Gans J (2006). Clinical features, complications, and outcome in adults with pneumococcal meningitis: a prospective case series. Lancet Neurol.

[15] Schut ES, Lucas MJ, Brouwer MC, Vergouwen MD, van der Ende A, van de Beek D (2012). Cerebral infarction in adults with bacterial meningitis. Neurocrit Care.

[16] Aronin SI, Peduzzi P, Quagliarello VJ (1998). Community-acquired bacterial meningitis: risk stratification for adverse clinical outcome and effect of antibiotic timing. Ann Intern Med.

[17] Bodilsen J, Dalager-Pedersen M, Schønheyder HC, Nielsen H (2016). Time to antibiotic therapy and outcome in bacterial meningitis: a Danish population-based cohort study. BMC Infect Dis.

[18] de Gans J, van de Beek D (2002). Dexamethasone in adults with bacterial meningitis. N Engl J Med.

[19] Tunkel AR, Hartman BJ, Kaplan SL, Kaufman BA, Roos KL, Scheld WM, Whitley RJ (2004). Practice guidelines for the management of bacterial meningitis. Clin Infect Dis.

[20] Leber AL, Everhart K, Balada-Llasat JM (2016). Multicenter evaluation of the BioFire FilmArray meningitis/encephalitis panel for detection of bacteria, viruses, and yeast in cerebrospinal fluid specimens. J Clin Microbiol.

[21] Shih RY, Koeller KK (2015). Bacterial, fungal, and parasitic infections of the central nervous system: radiologic-pathologic correlation and historical perspectives. Radiographics.

[22] Hasbun R, Abrahams J, Jekel J, Quagliarello VJ (2001). Computed tomography of the head before lumbar puncture in adults with suspected meningitis. N Engl J Med.

[23] Lesourd A, Magne N, Soares A (2018). Primary bacterial ventriculitis in adults, an emergent diagnosis challenge: report of a meningoccal case and review of the literature. BMC Infect Dis.

[24] Govindarajan S, Bivan S (2020). Viral and bacterial coinfection of the respiratory tract in a 10-month-old child. BMJ Case Rep.

[25] Hupp S, Förtsch C, Graber F, Mitchell TJ, Iliev AI (2022). Pneumolysin boosts the neuroinflammatory response to Streptococcus pneumoniae through enhanced endocytosis. Nat Commun.

[26] Deliran SS, Brouwer MC, van de Beek D (2025). Cerebrovascular complications in bacterial meningitis. Stroke Vasc Interv Neurol.

[27] Weller J, Enkirch JS, Lehmann F, Radbruch A, Klockgether T, Zimmermann J (2022). Early intracranial haemorrhage predicts poor clinical outcome in community-acquired bacterial meningitis. Front Neurol.

[28] Cook AM, Morgan Jones G, Hawryluk GW (2020). Guidelines for the acute treatment of cerebral edema in neurocritical care patients. Neurocrit Care.

[29] Tunkel AR, Glaser CA, Bloch KC (2008). The management of encephalitis: clinical practice guidelines by the Infectious Diseases Society of America. Clin Infect Dis.

[30] Lewis A, Kirschen MP, Greer D (2023). The 2023 AAN/AAP/CNS/SCCM pediatric and adult brain death/death by neurologic criteria consensus practice guideline: a comparison with the 2010 and 2011 guidelines. Neurol Clin Pract.

